# Neoantimon: a multifunctional R package for identification of tumor-specific neoantigens

**DOI:** 10.1093/bioinformatics/btaa616

**Published:** 2020-09-16

**Authors:** Takanori Hasegawa, Shuto Hayashi, Eigo Shimizu, Shinichi Mizuno, Atsushi Niida, Rui Yamaguchi, Satoru Miyano, Hidewaki Nakagawa, Seiya Imoto

**Affiliations:** Division of Health Medical Data Science, Health Intelligence Center, The Institute of Medical Science, The University of Tokyo, Tokyo 108-8639, Japan; Laboratory of DNA Information Analysis, Human Genome Center, The Institute of Medical Science, The University of Tokyo, Tokyo 108-8639, Japan; Laboratory of DNA Information Analysis, Human Genome Center, The Institute of Medical Science, The University of Tokyo, Tokyo 108-8639, Japan; Devision of Cancer Research, Center for Advanced Medical Innovation, Kyushu University, Fukuoka 812-8582, Japan; Division of Health Medical Data Science, Health Intelligence Center, The Institute of Medical Science, The University of Tokyo, Tokyo 108-8639, Japan; Laboratory of DNA Information Analysis, Human Genome Center, The Institute of Medical Science, The University of Tokyo, Tokyo 108-8639, Japan; Laboratory of DNA Information Analysis, Human Genome Center, The Institute of Medical Science, The University of Tokyo, Tokyo 108-8639, Japan; Laboratory for Cancer Genomics, RIKEN Center for Integrative Medical Sciences, Yokohama 230-0045, Japan; Division of Health Medical Data Science, Health Intelligence Center, The Institute of Medical Science, The University of Tokyo, Tokyo 108-8639, Japan

## Abstract

**Summary:**

It is known that some mutant peptides, such as those resulting from missense mutations and frameshift insertions, can bind to the major histocompatibility complex and be presented to antitumor T cells on the surface of a tumor cell. These peptides are termed neoantigen, and it is important to understand this process for cancer immunotherapy. Here, we introduce an R package termed Neoantimon that can predict a list of potential neoantigens from a variety of mutations, which include not only somatic point mutations but insertions, deletions and structural variants. Beyond the existing applications, Neoantimon is capable of attaching and reflecting several additional information, e.g. wild-type binding capability, allele specific RNA expression levels, single nucleotide polymorphism information and combinations of mutations to filter out infeasible peptides as neoantigen.

**Availability and implementation:**

The R package is available at http://github/hase62/Neoantimon.

## 1 Introduction

Recent technological advances in massively parallel sequencing have enabled the identification of genetic variants, e.g. single nucleotide variants (SNVs) and insertions or deletions (indels), in individual cancer patients. Furthermore, substantial evidence indicates that tumor-specific peptides that result from such variations can bind to a major histocompatibility complex (MHC) molecule and be presented to antitumor T cells on the surface of a tumor cell. Identification of such tumor-specific peptides, termed neoantigens, has been receiving increasing attention because of its numerous potential applications in cancer immunotherapy (Carreno *et al.*, 2015; Matsushita *et al.*, 2012; Mizuno *et al.*, 2018).

To identify the possible presence of neoantigens in individual tumor, we have to predict whether mutant peptides can bind to the patient’s human leukocyte antigens (HLAs). Several computational methods such as netMHCpan (Jurtz *et al.*, 2017) and MHCflurry (O’Donnell *et al.*, 2018) have been proposed to predict the binding capability, including binding affinity and percentage rank of affinity. To apply these methodologies, we need to determine the patient’s HLA types and prepare a list of tumor-specific peptides obtained from sequencing data. Designing such peptides requires not only mutation information, but also the reference sequences with their coding protein information because they are fractions of expressed mutant proteins. Even after the prediction of the binding capability of such mutant peptides, further classification filters should be applied to the selection, e.g. a comparison in binding affinity between wild-type and mutant peptides and the evaluation of allele-specific RNA expression levels.

To automate this process and easily identify tumor-specific neoantigens, some computational tools have been developed (Bjerregaard *et al.*, 2017; Hundal *et al.*, 2016). These tools greatly help to obtain predicted results; however, a few tools can use mutation data [such as variant call format (vcf) files] in a local analytical environment such as the R. In addition, the existing tools are applicable only for the prediction of HLA Class I binding and for handling of SNVs and indels at best, but not for the prediction of HLA Class II binding and handling of structural variants (SVs) and mutant RNA sequences such as fusion transcript. Moreover, they lack detailed considerations, e.g. reflecting SNVs on the frameshift regions and single nucleotide polymorphisms (SNPs) to generated peptides. To address these requirements, we developed an easy and multifunctional R package termed Neoantimon that can produce a list of candidate neoantigens (for HLA Class I and II) caused by SNVs, indels and SVs. It can automatically construct mutant peptides from vcf files or mutant RNA sequences and calculate their binding capability to the corresponding HLAs with some information for filtering. This tool has been used in the Mitochondrial Genome and Immunogenomics Working Group in the PanCancer Analysis of Whole Genomes (PCAWG) project (Mizuno *et al.*, 2018).

## 2 Implementation

### 2.1 Required input files

**Fig. 1.**
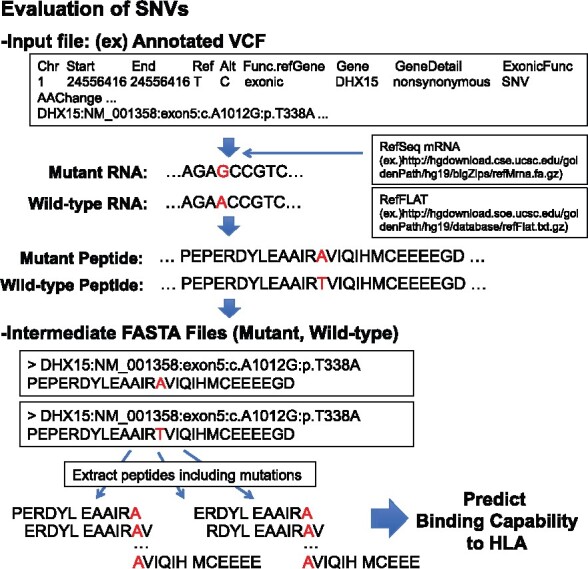
A sample procedure to generate mutant peptides from SNVs described in the input vcf file. Mutant and wild-type peptides are generated through the construction of the corresponding RNA sequences using Reference Sequence Database (RefSeq). Reference and alternative alleles, and wild-type and mutant amino acids are colored red.

**Fig. 2.**
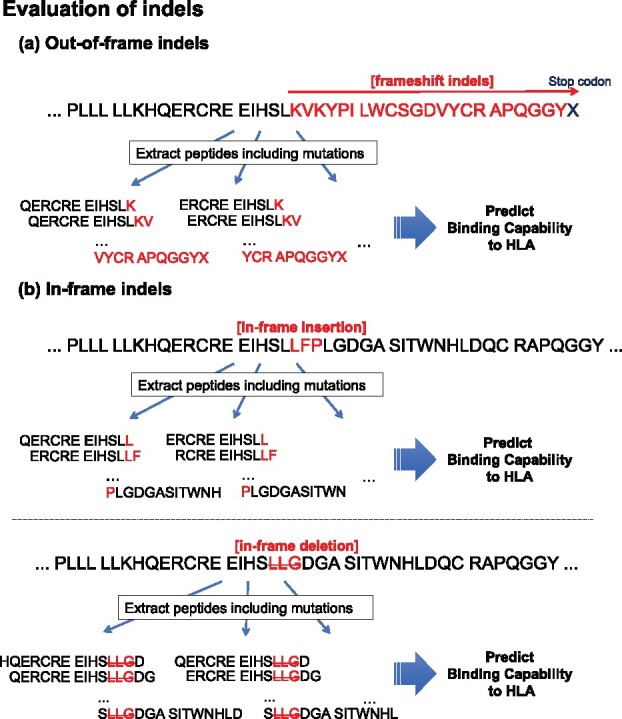
A sample procedure to generate mutant peptides from indels described in the input vcf file. In contrast to generate mutant peptides based on SNVs, indels are separated to (**a**) out-of-frame and (**b**) in-frame indels. In the former case, mutant peptides are generated from the mutation position to the stop codon. In the latter case, it is similar to the case of non-synonymous SNVs, as illustrated in Figure 1. The original peptide sequences are colored black, and mutant peptide and deleted peptide regions are colored red

**Fig. 3.**
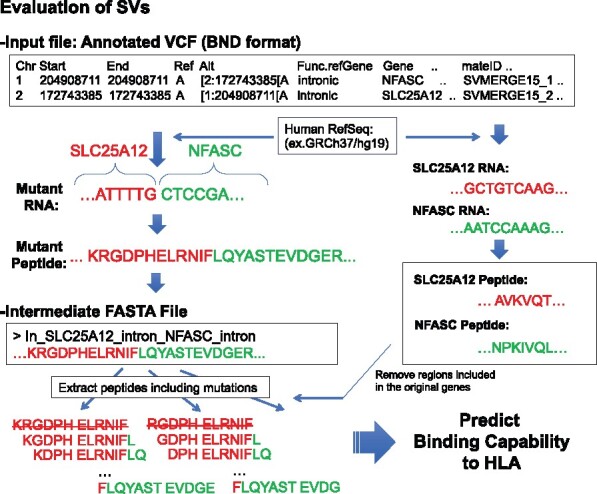
A sample procedure to generate mutant peptides from SVs described in the input vcf file following BND format. As same as indels, it can generate out-of-frame and in-frame potential fusion transcripts. By referring to the original protein sequences, peptides included in the original genes are removed for the evaluation. Here, RNA sequences and peptides generated from SLC25A12 and NFASC are colored red and green, respectively

**Fig. 4.**
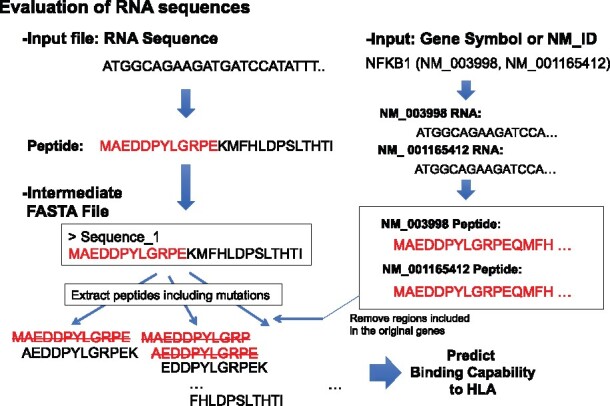
A sample procedure to generate mutant peptides directly from an RNA sequence. By referring to the original peptide sequences, peptides included in the original genes are removed for the evaluation. Here, peptides sequence included in NFKB are colored red. Note that, in this case, we assume that the input RNA sequence is a fusion transcript generated from NFKB

This package requires the two following inputs: (i) a text file storing gene sequence variations and (ii) a list of HLA types identified by, e.g. ALPHLARD (Hayashi *et al.*, 2018). (i) can be either one of the following files: (a) an annotated vcf file generated by ANNOVAR (Wang *et al.*, 2010) or Ensembl Variant Effect Predictor (VEP) (McLaren *et al.*, 2016), (b) non-annotated vcf file with using annotation option in this package or (c) a list of mutant RNA sequences in association with the corresponding gene symbols or NM IDs to filter out wild-type peptides. Only non-synonymous substitutions, in-frame and out-of-frame indels are extracted from SNVs and indels described in vcf files to generate mutant peptides. For the evaluation of potential fusion transcripts on the basis of SVs, vcf files must conform to the break-end (BND) format. A sample procedure to generate mutant peptides is illustrated in Figures 1–4.

### 2.2 Optional input files and considerations

**Fig. 5.**
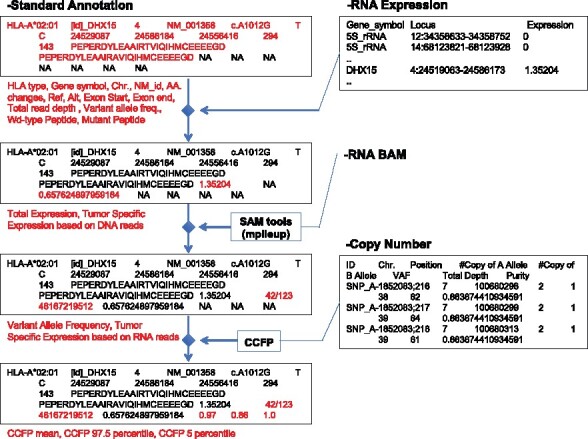
An explanation of attaching total and allele-specific RNA expressions, and CCFP. Additional information can be attached to the standard annotation in the output using (a) RNA expression data with and without (b) the corresponding Binary Alignment Map (BAM) file and (c) copy number variation data. Total and allele-specific RNA expression based on DNA alleles (VAF/total read depth × total RNA expression level), VAF and allele-specific RNA expression based on RNA sequences using samtools, and CCFP are attached given (a), (a) and (b) and (c)

Users can optionally provide (a) RNA expression data with and without (b) the corresponding Binary Alignment Map (BAM) file to attach total and allele-specific RNA expression levels and (c) copy number variation data to calculate the posterior probability distribution over cancer-cell fraction (CCFP) to evaluate tumor subclonality (Lohr *et al.*, 2014). Considering a somatic mutation observed in *a* of *N* sequencing reads on a locus of absolute somatic copy-number *q* in a sample of purity *α*, CCFP is calculated as *P*(*c*) ∝ Binom(*a*/*N*, *f*(*c*)), where f(c)=αc/2(1−α)+αq) and *c* corresponds to a uniform prior. They are attached to the output, as illustrated in Figure 5. Note that, (c) should be according to the output format of ASCAT (Allele-Specific Copy Number Analysis of Tumors) (Van Loo *et al.*, 2010) or include locus, #copy of Allele A and B, variant allele frequency (VAF), total read depth and tumor purity (VAF and total read depth can be automatically assigned from the input file).

**Fig. 6.**
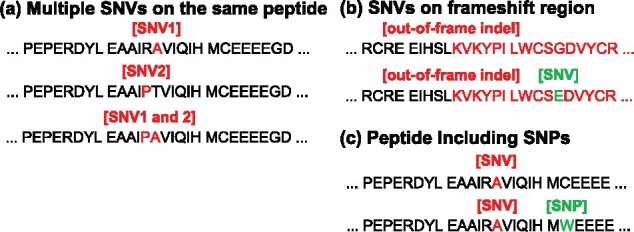
The consideration of the following cases; (**a**) multiple SNVs exist on the same peptide, (**b**) SNVs exist among the frameshift region generated by indels and (**c**) SNPs exist on the mutant peptide. In these cases, the package can optionally generate all patterns of mutant peptides and evaluate them

In addition, users can consider specific cases of existing SNPs on the mutant peptide by providing (c) SNPs data, and multiple SNVs on the same mutant peptides and among the frameshift region caused by indels. These cases are explained in Figure 6.

The comparison of the functions among pVACseq (Hundal *et al.*, 2016) and MuPeXI (Bjerregaard *et al.*, 2017), and our R package (neoantimon) is displayed in Table 1. One can install this package from a GitHub repository and use it in the R environment on Mac/Linux.

**Table 1.** A comparison table of the functions among pVACseq, MuPeXI and Neoantimon

	Neoantimon	pVACseq	MuPeXI
Input file format	vcf file	vcf file	vcf file
Variant annotation	YES	YES	YES
SNV support	YES	YES	YES
Indel support	YES	YES	YES
SV support	YES	NO	NO
RNA seq. support	YES	NO	NO
Output wild-type	YES	YES	YES
Total RNA exp.	Manual	Manual	Manual
Allelic RNA exp.	BAM required	BAM required	NO
Subclonal filter	YES	NO	NO
Multiple SNVs	YES	NO	NO
SNVs on frameshift	YES	NO	NO
SNPs integration	YES	NO	NO
Prediction software	netMHCpan/	netMHCpan/	netMHCpan
	MHCflurry	MHCflurry, etc.	

*Note*: ‘manual’ means that users manually upload RNA expression files.

### 2.3 Output files

**Fig. 7.**
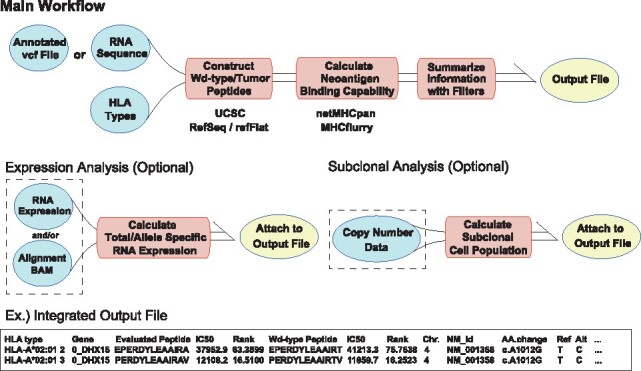
An overview of the input and output file information of the package. Green circles with and without dotted rectangles are optional and required inputs, respectively. Red rectangles and yellow circles are intermediate processes and the output files, respectively

This package generates FASTA files consisting of mutant and corresponding wild-type (for SNVs) peptides according to the RefSeq trasncript sequences, and an integrated output file including peptide–MHC binding capability estimated by NetMHCpan4.0 (Jurtz *et al.*, 2017) or MHCflurry (O’Donnell *et al.*, 2018), and NetMHCIIpan3.2 (Andreatta *et al.*, 2015). In the application to frameshift indels and potential fusion transcripts, all mutant peptides generated from the mutation position to the stop codon are constructed. The output file includes (i) the HLA type, (ii) gene symbol, (iii) wild-type (if exists) and mutant peptide sequences, (iv) their IC_50_ and percentages of rank affinity calculated by either NetMHCpan or MHCflurry (user selected), (v) chromosome number, (vi) NM_ID, (vii) amino acid changes (AAchages), (viii) reference and alternative alleles, (xi) exon start and end positions, (x) mutation position, (xi) total read depth and VAF, (xii) corresponding total and allele specific RNA expression (optional), (xiii) tumor subclonality represented by CCFP and the priority score defined in the next subsection. From the output file, users can extract a plausible set of peptides for neoantigen by setting any threshold for binding capability, RNA expressions and CCFP (such functions are also implemented). A simple overview of the package is illustrated in Figure 7.

### 2.4 Calculation of priority score

For the evaluation of generated mutant peptides, we propose priority scores to roughly predict their immunogenicity. Based on the previous research (Bjerregaard *et al.*, 2017), we calculate the priority scores *P_I_* and *P_R_* for using IC_50_ and percentage of rank affinity, respectively, as follows.

(1) $$P_{I}=[L_{I}(I_{m})f(A,E)][(1-2^{-M}L_{I}(I_{w})]C,$$

 

(2) $$P_{R}=[L_{R}(R_{m})f(A,E)][(1-2^{-M}L_{R}(R_{w})]C,$$

 

(3) f(A,E)=tanh(g(A)E),

 

(4) g(A)=tanh(5A),

 

(5) $$L_{I}(x)=\frac{1}{1+{\;\text{exp}\;}^{0.015(x-500)}},$$

 

(6) $$L_{R}(x)=\frac{1}{1+{\;\text{exp}\;}^{5(x-2)}},$$

 

(7) $$C=\frac{1}{1-{\;\text{exp}\;}^{-30(x-0.8)}},$$

where *I_m_* and *R_m_* are IC_50_ and percentage of rank affinity of the mutant peptide, respectively, and *I_w_* and *R_w_* are IC_50_ and percentage of rank affinity of the wild-type peptide, respectively. *E* is the expression level of the corresponding gene, *A* is the variant allele frequency, *M* is the number of mismatches between the mutant and wild-type peptides and *C* is the median value of CCFP. In contrast to the previous research (Bjerregaard *et al.*, 2017), the output does not include any peptide that perfectly matches to wild-type peptides. For the comparison, output file also includes the previous score.

## 3 Conclusions

We developed an R package generating candidate neoantigens from variety of mutations, i.e. SNVs, indels and SVs and mutant RNA sequences. In particular, this package can cover specific cases such as multiple SNVs on the same mutant peptide and among frameshift region, as explained in Figure 6, and include RNA expression and tumor subclonality data to filter-out implausible peptides for neoantigen. Thus, as compared in Table 1, it enables us to more finely select the candidate neoantigens beyond previously developed platforms. For the use of tumor immunotherapy, it can be attractive because it requires a great deal of cost to evaluate candidate mutant peptides to be neoantigen. Thanks to our package, one can easily complete the evaluation processes of candidate neoantigens by integrating several information. The package, documentation and sample analysis are available at http://github/hase62/Neoantimon, and an analysis result in PCAWG project is also available (Mizuno *et al.*, 2018).

## Funding

SI received Grant-in-Aid for Scientific Research(B) Grant Number 18H03328 from Japan Society for the Promotion of Science (https://www.jsps.go.jp/ english/).

*Financial Support*: none declared.

*Conflict of Interest*: none declared.
